# NAC Transcription Factors in Senescence: From Molecular Structure to Function in Crops

**DOI:** 10.3390/plants4030412

**Published:** 2015-07-13

**Authors:** Dagmara Podzimska-Sroka, Charlotte O’Shea, Per L. Gregersen, Karen Skriver

**Affiliations:** 1 Department of Genetics and Biotechnology, Aarhus University, Forsøgsvej 1, Slagelse DK-4200, Denmark; E-Mails: dagmara.podzimska-sroka@mbg.au.dk (D.P.-S.); per.gregersen@mbg.au.dk (P.L.G.); 2 Department of Biology, University of Copenhagen, 5 Ole Maaloesvej, Copenhagen DK-2200, Denmark; E-Mail: coshea@bio.ku.dk

**Keywords:** NAC transcription factor, domain structure, senescence, abiotic stress, gene regulatory network, crop breeding

## Abstract

Within the last decade, NAC transcription factors have been shown to play essential roles in senescence, which is the focus of this review. Transcriptome analyses associate approximately one third of Arabidopsis NAC genes and many crop NAC genes with senescence, thereby implicating NAC genes as important regulators of the senescence process. The consensus DNA binding site of the NAC domain is used to predict NAC target genes, and protein interaction sites can be predicted for the intrinsically disordered transcription regulatory domains of NAC proteins. The molecular characteristics of these domains determine the interactions in gene regulatory networks. Emerging local NAC-centered gene regulatory networks reveal complex molecular mechanisms of stress- and hormone-regulated senescence and basic physiological steps of the senescence process. For example, through molecular interactions involving the hormone abscisic acid, ArabidopsisNAP promotes chlorophyll degradation, a hallmark of senescence. Furthermore, studies of the functional rice ortholog, *OsNAP*, suggest that NAC genes can be targeted to obtain specific changes in lifespan control and nutrient remobilization in crop plants. This is also exemplified by the wheat *NAM1* genes which promote senescence and increase grain zinc, iron, and protein content. Thus, NAC genes are promising targets for fine-tuning senescence for increased yield and quality.

## 1. Introduction

Plant senescence has been extensively reviewed by a number of authors (see e.g., [1,2,3,4,5,6,7,8,9,10]), and the general understanding of senescence in the developmental process is well established. Senescence in plants corresponds to the age-dependent programmed degradation and degeneration process of cells, tissues, organs, or the entire organism, leading to death [11], with the special role of rescuing nutrients for still living parts of the plant. For trees and other perennial plants, leaf senescence is illustrated by the spectacular changes in leaf color that occur in the autumn in temperate regions of the world. Similar processes are observed in annual plants such as grain crops when they turn from green to golden as the grain ripens before harvest [4].

Leaf senescence is not a passive and unregulated degeneration process. During senescence, leaf cells undergo dramatic changes in structure, metabolism, and gene expression in an orderly manner. The earliest and most significant change in the cell structure is breakdown of chloroplasts, which contain most of the leaf protein [11]. The mitochondria and nucleus remain intact until the last stages of senescence [6]. Metabolic changes during leaf senescence include loss of photosynthetic activities and hydrolysis of macromolecules, such as proteins, membrane lipids, and nucleic acids [12]. In annual plants, these hydrolyzed molecules are relocated to developing seeds and fruits. In trees, the nutrients that are degraded during leaf senescence are stored in stems or roots and are utilized later for the development of new leaves or flowers [12].

The regulation of leaf senescence depends on the developmental age of plants but is also influenced by various internal and environmental factors [11]. The environmental stresses that effect leaf senescence include nutrient deficiency, salinity, extremes of light or temperature, radiation, drought, pathogen infection and the presence of toxic materials in the air, water, or soil [11,13,14]. Related to this, reactive oxygen species (ROS) also play a significant role in the senescence process [15]. Internal factors associated with senescence include age, reproductive development, and phytohormones [16].

Hormonal regulation of leaf senescence has been studied for decades, and the effect of plant hormones on senescence has been reported for various plant species [17]. The role of hormones in regulation of leaf senescence is becoming evident through characterization of genetic mutants and global gene expression analysis, providing important molecular information about how the hormonal signaling pathways lead to changes in gene expression patterns during leaf senescence [11]. It is also well known that senescence is accelerated by brassinosteroids, abscisic acid (ABA), ethylene, jasmonic acid (JA), methyl jasmonate (MeJA), and salicylic acid (SA), and delayed by auxin, cytokinins and gibberellic acid (GA) [18].

The best evidence for hormonal involvement in the control of senescence has been demonstrated for ABA, ethylene, and cytokinins. ABA plays a major role in various aspects of plant growth and development, including regulation of seed maturation and germination, as well as adaptation to environmental stresses [19]. Dramatic increases in the endogenous ABA level and up-regulation of genes associated with ABA-signaling have been observed during leaf senescece in many plants, including tobacco (*Nicotiana tabacum*) [20], oat (*Avena sativa*) [21], rice (*Oryza sativa*) [22], maize (*Zea mays*) [23] and *Arabidopsis thaliana* [24]. Moreover, exogenously applied ABA and salinity induce expression of several senescence-associated genes (SAGs) known to accelerate leaf senescence [25,26,27], suggesting substantive mechanisms that connect leaf senescence and ABA and abiotic stress signaling [28]. Furthermore, ABA is involved in response to abiotic stresses such as drought, high salinity, and low temperatures that promote leaf senescence [29]. Therefore, it seems clear that ABA acts as a key positive regulator of leaf senescence.

Ethylene is essential for many plant processes such as seed germination, seedling development and fruit ripening, but it has also been shown to have a role in the regulation of the onset of senescence [30]. Thus, exposure of Arabidopsis plants to ethylene induces premature yellowing of the oldest leaves [31], and the leaves of the two ethylene-insensitive mutants of *Arabidopsis thaliana*, *ethylene response 1* (*etr1*) and *ethylene-insensitive 2* (*ein2*), showed delayed onset of leaf senescence [32,33]. Furthermore, certain Arabidopsis mutant lines with delayed leaf senescence phenotype turned out to have defects in genes involved in ethylene biosynthesis and the ethylene signaling pathway [4]. However, in these cases senescence eventually progresses normally once the process has begun [4]. Thus, it has been concluded that ethylene is not essential for the occurrence of senescence and that it can only modulate the timing of leaf senescence [34]. In addition, the effect of ethylene on the induction of leaf senescence was shown to be under the direct influence of age [31]. Ethylene can only promote senescence in leaves that have reached a defined age and therefore young leaves treated with ethylene do not senesce [4].

It has been known for many years that cytokinins stongly influence retardation of leaf senescence [35], and several reports have illustrated the importance of cytokinin in the control of senescence [4]. The signal that initiates the onset of developmental senescence appears to be controlled by cytokinin levels [36,37]. In accordance with this, treatment with cytokinin can delay leaf senescence [37,38], while decrease in the level of cytokinin can lead to accelerated senescence [39].

Senescence has been intensively studied in the model plant Arabidopsis [4,25,27,40,41,42,43], whereas the molecular mechanism driving leaf senescence in crop plants is still limited. Increasing our knowledge about the senescence process (e.g., in cereals) could lead to the development of tools for the breeding of new varieties with increased yield, grain quality and nutrient efficiency [44,45]. One approach to achieve this is to focus on crop plants with delayed senescence (stay-green phenotype) [46,47] or by making the senescence process more robust towards abiotic stress [44]. Due to economic considerations, much of this research has focused on senescence in wheat (*Triticum aestivum*) [44], barley [45,48,49,50,51], rice [28], and cotton (*Gossypium hirsutum*) [52,53].

Particular interests lie in the identification of regulatory genes encoding transcription factors and signaling components that could be responsible for the onset of senescence [29]. Analysis of transcription factor genes has indicated that there is overlap between age-dependent senescence and senescence induced by different factors [54]. These findings suggest that complex regulatory networks function in leaf senescence processes. Here, we review the current knowledge of no apical meristem (NAM), ATAF1/2, cup-shaped cotyledon2 (CUC2) (NAC) transcription factor association with and function in senescence by relating NAC gene expression and structure to specific molecular interactions and presenting emerging local senescence-associated gene regulatory networks (GRNs) with central NAC genes and proteins. This reveals significant physiological functions of NAC transcription factors of putative use in future breeding for improved food quality.

## 2. NAC Transcription Factors in Senescence

Transcriptome studies indicate that a large number of genes from the NAC family are associated with the senescence processes [25,40,43,44,45]. The NAC gene family is one of the largest plant-specific transcription factor families, and members of the family are involved in various plant processes, including plant development, cell division, wood formation, and stress responses (for review see [55,56,57]), and as this review will show, NAC genes and proteins also play a crucial role in senescence. A significant functional role in leaf senescence has so far been demonstrated for e.g., *Arabidopsis thaliana NAC-like*, *activated by apetala3/pistillata* (*AtNAP*; *ANAC029*) [58], *Oresara1* (*ORE1*; *ANAC092*; *AtNAC2*) [59], *Oresara sister1* (*ORS1*; *ANAC059*) [60], *Jungbrunnen1* (*JUB1*; *ANAC042*) [61], *Vascular-related NAC-domain interacting* (*VNI2*; *ANAC083*) [62], *ANAC016* [63] and *Arabidopsis thaliana activating factor1* (*ATAF1*; *ANAC002*) [64]. Overexpression of *AtNAP* [58], *ORE1* [59,65], *ORS1* [60], *ANAC016* [63], and *ATAF1* [64] resulted in precocious senescence, whereas blocking the function of these genes delayed senescence suggesting that they function as nonredundant positive regulators of senescence in Arabidopsis. In contrast, *JUB1* and *VNI2* are negative regulators of leaf senescence [61,62]. Additionally, the closely related Arabidopsis NAC transcription factors *ANAC019*, *ANAC055*, and *RD26* (*ANAC072*) have been associated with senescence due to their senescence-induced expression patterns [27] and their position in senescence-associated GRNs [66]. In plants other than Arabidopsis, RNA interference (RNAi) studies of wheat *Gpc-B1*, encoding the NAC protein NAM-B1, resulted in delayed leaf senescence and significant reduction in grain zinc, iron, and protein content [67], and in rice, *Os*NAP is a positive regulator of senescence with an effect on the grain filling period and grain yield [28].

### 2.1. Arabidopsis NAC Gene Expression during Senescence

**Figure 1:**
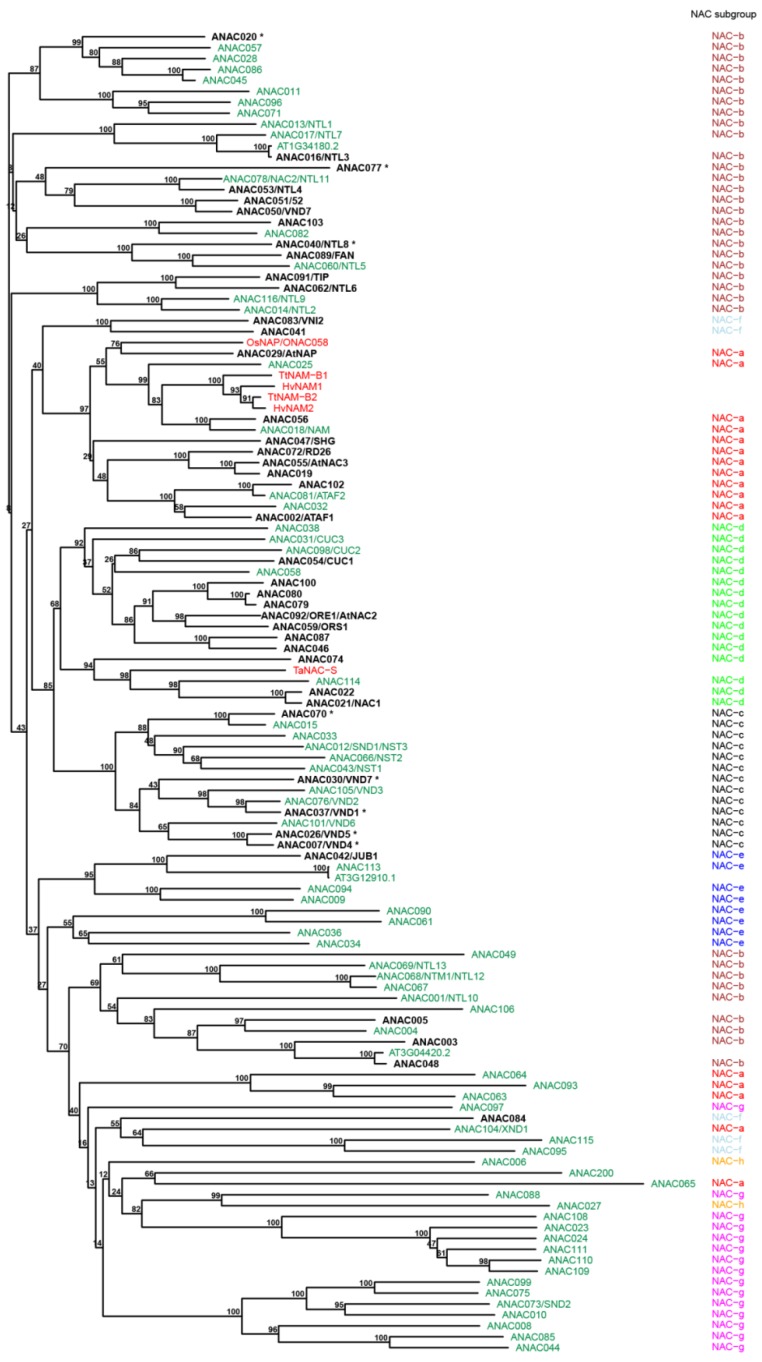
Phylogenetic presentation of NAC proteins. For Arabidopsis, the NAC proteins are shown in green with senescence-associated proteins highlighted in black and bold. Asterisks indicate down-regulation during senescence. For barley, wheat, and rice, only the senescence-associated proteins/genes discussed with respect to functional role in the text are shown in the tree. These proteins are shown in red. The tree was based on only the N-terminal NAC domain of the proteins and it was constructed using ClustalX [71] and the R add-on package ape [72]. The subfamily classification is based on the system proposed by Shen *et al.* [73].

Extensive transcriptome analyses have revealed that transcription factors from several different families are differentially expressed during leaf senescence [25,27,41,43], and NAC genes are well-represented among these [27,43]. Already in 2008, Balazadeh *et al.* [43] identified NAC genes, which were differently expressed during Arabidopsis leaf growth and the beginning of senescence. Among these, *AtNAP*, *ORS1*, and *ORE1*, which play significant roles in senescence [58,59,60,65], were up-regulated during senescence (Figure 1). Additional up-regulated NAC genes included *ANAC003*, *ANAC019*, *ANAC021/22* (*NAC1*), *SPEEDY HYPONASTIC GROWTH* (*SHYG*; *ANAC047*) [68], *ANAC048*, *ANAC055*, *ANAC056* (*NARS1*), *RD26* (*ANAC072*), *ANAC074*, *ANAC079/80*, and *ANAC100*. Fewer NAC genes, ANAC007 (*VND4*), *ANAC020*, *ANAC026* (*VND5*), *ANAC030* (*VND7*), and *ANAC037* (*VND1*) were down-regulated during the process. In a study focusing on the ORE1 regulatory network, *ANAC041*, *ANAC054* (*CUC1*), *ANAC084*, and *ANAC083* (*VNI2*) were suggested to be additional NAC genes up-regulated during senescence [65]. Later, approximately 35 NAC genes were shown to be up-regulated during leaf senescence (Figure 1), and they were generally significantly up-regulated already early, 19–21 days after sowing (DAS), during the process [27]. Also in this study, only a few NAC genes (*NTL8* (*ANAC040*), *ANAC070*, *ANAC077*, and *VND7*) were shown to be down-regulated. *AtNAP*, *ORE1*, and *VNI2* were significantly up-regulated already 21 DAS, and *ORS1* was up-regulated 23 DAS. Several JA-related genes were also up-regulated 23 DAS, and although ABA-levels peaked later during the process, several ABA-responsive NAC genes (*RD26*, *ANAC019*, *FAN* (*ANAO89*), *ANAC087*, *ANAC055*) were among the genes up-regulated already 25 DAS (*TIP* (*ANAC091*), *ANAC016* (*NTL3*), and *ANAC103* in addition to the ABA-responsive NAC genes). This may suggest coordination of JA- and ABA-responses involving NAC genes early during senescence [27]. In accordance with the significant association of NAC transcription factors with senescence based on the expression analysis, binding sites (BSs) for NAC proteins [69] were overrepresented in genes from several clusters sharing senescence dependent expression profiles [27]. For examples, the NAC-BS [69] was overrepresented in clusters 41 and 42, for which no or only weak up-regulation of gene expression was detectable until 13 DAS, and cluster 45 which showed up-regulation from the beginning of leaf development. Cluster 41 contains *ANAC003*, *NTL4* (*ANAC053*), and *ANAC084*, cluster 42 contains *ANAC055*, *ANAC080*, *ANAC087*, and *ANAC102*, and cluster 45 contains *ANAC046*, *SHG*, *ANAC052*, *VNI2*, and *ORE1*. Since co-expression occurs among transcription factors and their target genes [70], the genes with NAC-BSs are likely to be directly regulated by NAC transcription factors during senescence.

NAC gene expression during senescence has also been analyzed under perturbed conditions. Thus, the effect of abiotic stresses, in particular drought, salt stress, and wounding, on the expression of senescence-related NAC genes was analyzed several years ago [43]. For *ANAC019*, *ANAC055*, and *RD26* induction by salt stress was severe, whereas drought and wounding affected their expression much less. More recently, transcriptional profiling of Arabidopsis leaves during salt-induced senescence was compared with transcriptomes from leaves during developmental senescence and leaves exposed to the reactive oxygen species (ROS) H_2_O_2_ [26]. The ABA-responsive *cis*-acting element CACGTGT was significantly over-expressed in genes up-regulated in all three cases. Among these were five ABA-inducible NAC genes, *AtNAP*, *ANAC055*, *ANAC062* (*NTL6*), *RD26*, and *ANAC102*.

### 2.2. Crop NAC Gene Expression during Senescence

In 2007, Gregersen and Holm [44] investigated the changes in gene expression in wheat during flag leaf senescence. This was the first transcriptome analysis of the senescence process in cereal species. The results indicated that several different functional categories of genes, including regulatory components, were differentially expressed in wheat during flag leaf senescence. Among the identified transcription factors only three members of the NAC family, *Geminivirus Rep A-binding 2* (*GRAB2*), and orthologs of *OsNAC3* and *OsNAC6*, showed increased expression during senescence. Orthologs of these genes had previously been suggested to be associated with senescence in other plant species [25,27].

Later, Christiansen *et al.* [50] characterized 48 members of the barley (*Hordeum vulgare* L*.*) NAC gene family. The phylogenetic classification of the barley NAC genes (*HvNACs*) was performed according to the system proposed by [73] based on the alignment of the conserved NAC domain of e.g., rice, Arabidopsis, *Brachypodium distachyon*, and barley NAC genes. The NAC genes were distributed into eight distinct NAC subfamilies, from NAC-a to NAC-h (Figure 1), and each subfamily was divided into several subgroups based on the homology of the C-terminal motifs. Identified barley NAC (*HvNAC*) genes were characterized by their expression profile across different barley tissues and following hormone treatments. Correlating these expression profiles with functional information available for phylogenetically related NAC genes from other species resulted in the identification of putative regulators of several important plant processes in barley. Three barley *NAC* genes (*HvNAC005*, *HvNAC027*, and *HvNAC029/HvNAM1*) were identified as putative senescence regulators. They were up-regulated in senescing flag leaf, old ear, and late dough grain. Both *HvNAC005* and *HvNAC027* cluster with *AtNAP* (*ANAC029*) of subfamily NAC-a, a well-known positive regulator of senescence [58]. *HvNAC005* and *HvNAC027* were also up-regulated after ABA treatment [50]. *HvNAC0029/HvNAM1* is an ortholog of *TaNAM-B1*, also from subfamily NAC-a, involved in nutrient remobilization during senescence [67].

Recently, Christiansen and Gregersen [45] presented a list of 15 barley NAC genes representing putative regulators of senescence and biotic stress related traits affecting the productivity of cereal crop plants. Based on the gene expression patterns, the barley *NAC* genes were divided into three groups. The first group represents one gene, *HvNAC026*, which was strongly up-regulated in senescing flag leaves. It encodes a close homolog of *AtXND1* (*ANAC104*), a subfamily NAC-a NAC protein known as a putative negative regulator of xylem development in Arabidopsis [74], which however has not been described as involved in senescence. The second group contains the five closely related NAC-a genes, *HvNAC005*, *HvNAC023*, *HvNAC027*, *HvNAC029/HvNAM1*, and *HvNAC030/HvNAM2*, with early up-regulation and with a tendency to be down-regulated towards the later stages of senescence. The third group comprises genes which were highly expressed during the latest stage of flag leaf senescence, exemplified by the three NAC-d genes *HvNAC013*, *HvNAC022*, and *HvNAC025.* Seven models of palindromic NAC-BSs were used to screen available barley promoter sequences of barley SAGs. The analysis revealed 71 senescence-associated genes with a significant over-representation of the NAC-BSs, suggesting possible regulation of these SAGs by barley NAC transcription factors. Moreover, co-regulation studies showed that genes possessing the motifs in their promoter in general were highly co-expressed with members of the NAC gene family [45].

In rice, a suppression subtractive hybridization library was constructed to analyze for differentially expressed genes during the onset of flag leaf senescence [75]. Only one member of the NAC gene family, NAC-domain-containing protein 2, showed significant up-regulation in early senescent flag leaves [75]. More recently, microRNAs (miRNAs) and their putative target genes of two super hybrid rice varieties, Nei-2-You 6 (N2Y6, age-resistant rice) and Liang-You-Pei 9 (LYP9, age-sensitive rice), were analyzed by high-throughput sequencing [76]. Six miRNA families, osa-miR159, osa-miR160, osamiR164, osa-miR167, osa-miR172, and osa-miR1848, were shown to be associated with the rice leaf senescence processes through hormone signaling pathways. Four members of the miR164 family (osa-miR164a, osa-miR164b, osa-miR164d, and osa-miR164e) were found to target *NAC1* (*ONAC15*; *Os07g48550.1*) and *NAC21/NAC22* (*ONAC104*; *Os08g10080.1*). In leaves at early, middle, and late stages of grain filling, the expression level of osa-miR164a, osa-miR164b, osa-miR164d, and osa-miR164e was significantly higher in N2Y6 than in LYP9, and resulted in decreased expression of *NAC1* and *NAC21/NAC22*, by which leaf senescence of N2Y6 could be delayed. This may indicate one of the mechanisms of osa-miR164 mediated senescence-resistance in rice [76].

In cotton (*Gossypium hirsutum* L.), several NAC genes (*GhNACs*) were isolated and characterized in relation to leaf senescence and diverse abiotic stresses [52,77,78]. Phylogenetic comparisons to Arabidopsis, *Brassica napus*, rice, and citrus NAC proteins indicated that ten identified cotton NAC genes (*GhNAC8-GhNAC17*) could be classified into three distinct groups [52]. *GhNAC8*, *GhNAC10*, *GhNAC11*, and *GhNAC17* clustered together with Arabidopsis *NAM*, *GhNAC9* and *GhNAC12* clustered together with *AtNAP*, and *GhNAC13*, *GhNAC14*, *GhNAC15*, and *GhNAC16* clustered together with *ATAF1*. All three Arabidopsis NAC genes belong to subfamily NAC-a, which contains several senescence- and stress-associated NAC proteins (Figure 1) [27,43,57,79,80]. The closest homolog of *GhNAC9*, *GhNAC10*, and *GhNAC12* are *AtNAP*, *ANAC084* and *ORE1*, respectively, which are either associated with senescence (*ANAC084*) or well known for their regulatory role in leaf senescence (*AtNAP* and *ORE1*) [58,65]. The rest of the *GhNACs* genes were homologs of stress-responsive genes from cotton, rice, Arabidopsis, and Brassica [52]. All the characterized *GhNACs* were significantly up-regulated in senescing leaves except *GhNAC13*, which was down-regulated during the process. Moreover, the *GhNACs* displayed differential expression patterns during natural and induced leaf senescence. The expression of the *GhNAC* genes in response to several hormones was also examined, and several of these were up-regulated after ABA- and ethylene-treatment in a time-dependent manner. Thus, expression of *GhNAC8*, *GhNAC12*, and *GhNAC14* was gradually induced by ABA and reached a peak after 48 h of treatment. The highest expression levels of the *GhNAC13*, *GhNAC15*-*GhNAC17* genes were observed after 12 or 24 h of ABA-treatment followed by a decline. *GhNAC10* and *GhNAC16* were highly up-regulated in response to ethylene, while *GhNAC8*, *GhNAC9*, and *GhNAC17* were not significantly affected by the ethylene treatment. These results suggest a putative role of the cotton *GhNAC* genes in leaf senescence, possibly integrated with ABA or ethylene [52]. More recently, extensive global analysis of the cotton transcriptome revealed that more than 40 members of the NAC gene family were significantly up-regulated at various times during senescence, indicating their putative role in cotton leaf development and senescence [53].

In conclusion, based on transcriptome analyses, the expression of approximately one third of the Arabidopsis NAC genes [79] is affected by the senescence process (Figure 1), and the major part of these are up-regulated during the process. Although the association of crop NAC genes with senescence is not characterized as well as for Arabidopsis, transcriptomic studies have identified a number of senescence-associated NAC genes from wheat, barley, and cotton. As in the case of Arabidopsis, these genes mainly belong to subfamily NAC-a, NAC-b, and NAC-d, but senescence-associated NAC genes are spread over the complete phylogenetic NAC tree (Figure 1). In addition, based on the large scale analyses, several of the senescence-associated genes from both Arabidopsis and crop plants are associated with hormone signaling, mainly ABA-signaling, and abiotic stress responses. This in itself is suggestive of cross talk between age-dependent and environmental-influenced senescence mechanisms.

## 3. NAC Proteins and Senescence Associated Interactions

In order to understand and engineer NAC proteins, detailed knowledge on the molecular level is needed. NAC proteins contain an N-terminal dimerization and DNA binding domain, the NAC domain, and diverse C-terminal transcription regulatory domains involved in specific interactions with DNA and other proteins.

### 3.1. The NAC Domain and DNA Binding

**Figure 2:**
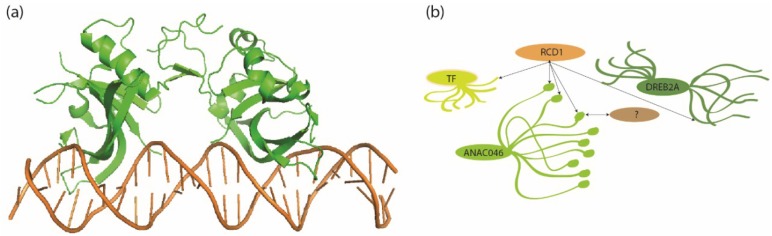
Structure and interactions of NAC protein domains. (**a**) Model of dimeric ANAC019 NAC domain (shown in green) bound to target DNA (PDB accession code 3SWP) [82]; (**b**) Interactions of senescence associated ANAC046 and RCD1. ANAC046 has a folded N terminal DNA binding NAC domain (here shown as an ellipse and in (**a**) as a cartoon) and an intrinsically disordered C-terminal transcription regulatory domain (shown as different conformers). ANAC046 uses its C-terminal molecular recognition feature (shown as a small ellipse in the different conformers) for interactions with RCD1, likely in competition with other yet unidentified proteins (shown by “?”). The interactions are indicated by arrows. RCD1 in turn is a hub protein and interacts with multiple transcription factors (shown by arrows), including ANAC046 and DREB2A, by exploiting the flexibility of their intrinsic disorder as shown by different conformers. The transcription factors may compete for the same binding site in RCD1 as shown by the common origin of the arrows on RCD1. The figure is based on work by Jaspers *et al.* [91] and O’Shea *et al.* [92]

The tertiary structure of the NAC domain from Arabidopsis ANAC019 has been determined by X-ray crystallography. The domain forms a β sheet surrounded by α helices [81] and inserts an outer β strand with a highly conserved amino acid sequence into the major groove of the DNA binding site [82,83] (Figure 2a). Several recent studies have addressed binding by this domain to synthetic oligonucleotides and target promoters using different experimental approaches. Lindemose *et al.* [84] used protein binding microarrays to define DNA binding specificities for twelve NAC proteins. This revealed that the NAC proteins can be separated into clusters based on their DNA binding preferences and that the clusters largely match the phylogenetic relationship of the proteins. Cluster I, which contains senescence-associated ANAC019, ANAC055, ORE1, *At*NAP, and ATAF1, showed a binding preference for the NAC-BS consensus model T[G/A] CGT [69] (Table 1). In accordance with this, the direct target genes of ANAC019, *VEGETATIVE STORAGE PROTEIN1* (*VSP1*) and *EARLY RESPONSIVE TO DEHYDRATION STRESS 1* (*ERD1*), the direct target genes of *At*NAP, *SAG113* and *ABSCISIC ALDEHYDE OXIDASE 3* (*AAO3*), and the direct target genes of ATAF1, *ORE1*, *Golden2-like 1* (*GLK1*), 9-*cis-epoxycarotenoid dioxygenase* (*NCED3*) and the ABA-transporter *ABCG40*, contain at least one copy of the NAC-BS core CGT in the region bound by the NAC protein (Table 1) [64,85,86,87,88,89]. As apparent from *At*NAP and ATAF1 target gene binding, a single NAC-BS is sufficient for binding *in vivo* [86,87,88]. However, in accordance with NAC proteins being dimers [81], a bipartite NAC-BS was selected and defined for ORS1 [60], ATAF1 [64], and JUB1, and the target gene of JUB1, *DEHYDRATION RESPONSIVE ELEMENT-BINDING PROTEIN2A* (*DREB2A*), contains two palindromic NAC-BS core motifs in the region bound by JUB1 (Table 1) [61]. This suggests that both monomers of JUB1 bind specific sequences in the *DREB2A* promoter enhancing both affinity and specificity of binding. For ATAF1, two binding sites, one of which is bipartite, most likely are present in the *NCED3* promoter (Table 1) [64,88].

In the protein binding microarray analysis, cluster 3, containing NTL6 and NTL8, showed specificity for the TT[A*/*C*/*G] CTT consensus model [84], which does not resemble the NAC-BS. Interestingly, despite of this, senescence-associated NTL4, closely related to NTL6 and NTL8 [79] (Figure 1), also bound to the NAC-BS core in the *Arabidopsis thaliana* respiratory burst oxidase (*Atrboh*) A, C, and E target genes, which encode ROS biosynthetic proteins [90]. This sequence was also represented by k-mers bound to the NTL6 and NTL8 NAC proteins, but with low enrichment scores [84]. So, for all known direct target genes of senescence-associated NAC proteins the NAC-BS core CGT is part of the binding sequence. Therefore, different variations, e.g., palindromic versions, of the NAC-BS (Table 1), can be used to identify direct NAC target genes from co-expressed senescence-associated genes. This strategy has already been used in large scale studies of NAC genes in senescence [27,45,65].

**Table 1:** DNA and promoter sequences bound by senescence associated NAC proteins.

NAC	Synthetic DNA/Target Gene	Ref.
ANAC019	5′-AARTTACGTR-3′	[84]
ANAC019 *	5′-TGCGTGGACATGCGTGGACATGCGTGGACATGCGTGGACATGCG-3′ (*VSP1*) ^**^	[85]
ANAC019	5′-ACATGCGTGT(7n)GA-3′ (*ERD1*)	[89]
ANAC055	5′-AARTTACGTR-3′	[84]
*At*NAP	5′-GTTACGTAT-3′	[84]
*At*NAP	5′-. . .CACGTAAGT…-3′*** (52 bp of *SAG113*)	[86]
*At*NAP	5′-AGATGTGCGTGAAAGAGGCGCAACTATAAGAG-3′ (*AAO3*)	[87]
ATAF1	5′-AAGTTACGTA-3′	[84]
ATAF1	5′-…TAAATTGCGTGCGT…-3′ (131 bp fragment of *NCED113*)	[88]
ATAF1	5′-VDHVnnYRR(5-6n)YACGnMWSK-3′ and 5′-RRWnnCGTR(5-6n)DACGTMWHD-3′	[64]
ATAF1	5′-…GTTGCCTAACAATCTACGCATCT…-3′ (40 bp fragment of *ORE1*)	[64]
ATAF1	5′-…GACCAGTAGTCCTTTTACGAAAGT…-3′ (40 bp fragment of *GLK1*)	[64]
ATAF1	5′-…GACACTAATATAAACTACGTACC…-3′ (40 bp fragment of *NCED3*)	[64]
ATAF1	5′-…AGATGCGTGGGAGGCTTGGGGAC…-3′ (40 bp fragment of *ABCG40*)	[64]
ORS1	5′-RCGTR(4-5n)RYACGCAA-3′	[60]
JUB1	5′-TGCCGT7nACG-3′and 5′-TGCCGTNNNNNNNCCGC-3′	[61]
JUB1	5′-…GATGCCGTTAGAGACACG…-3′ (40 bp of *DREB2A*)	[61]
NTL4	5′…TTATGTCGTACGAAA…3′ (*AtrbohC*)	[90]
NTL4	5′-…TACGTGGCGTAATCC…-3′ (*AtrbohE*)	[90]

*: And ANAC055 and RD26; ^**^: NAC-BS core motif underlined; ***: … shows extension of the sequence; W = A, T; K = G, T; R = A, G; V = A, C, G; M = A, C; D = T, G, A; Y = C, T; H = A, C, T; S = C, G.

### 3.2. The Transcription Regulatory Domain and Protein Interactions

In contrast to the structurally well-defined DNA binding domain of transcription factors, the transcription regulatory domains have a propensity for flexible protein segments that do not form fixed tertiary structures. This feature is known as protein intrinsic disorder (ID) [93,94]. ID in NAC proteins was first experimentally addressed for barley senescence-associated *Hv*NAC005 and *Hv*NAC013. The transcription regulatory domains of these NAC proteins were shown by biophysical analysis to be mostly disordered [95]. Furthermore, *Hv*NAC013 interacted with barley RADICAL INDUCED CELL DEATH1 (RCD1). More recently, the transcription regulatory domains of three senescence-associated Arabidopsis NAC proteins, ANAC019, *At*NAP, and ANAC046, were also shown to be disordered [92]. ANAC0046, the closest Arabidopsis homolog of *Hv*NAC013, also interacted with RCD1. The interaction took place through the only region with structure propensity in the ANAC046 transcription regulatory domain, a so-called molecular recognition feature [96], which was also predicted to be the interactions site of ANAC046. It was suggested that the ANAC046 transcription regulatory domain functions as an entropic chain with a terminal hot spot for interactions with RCD1 and other proteins (Figure 2b) [92]. RCD1 is involved in a variety of stress and developmental responses [97], including senescence. The *rcd1* mutant showed altered programmed cell death (PCD) in response to ROS [98] and accelerated senescence combined with normal expression of senescence-related genes, suggesting that senescence in *rcd1* plants is an atypical process [99]. RCD1 as a so-called hub protein interacted with numerous proteins, predominantly transcription factors [91]. ANAC046 and DREB2A, and its close paralogs DREB2B and DREB2C, were among the interactions partners. The interaction between RCD1 and DREB2A was mediated by a short sequence motif in an overall disordered region in DREB2A [99,100]. A splice variant of DREB2A, which lacks this motif, accumulated during heat stress and senescence, suggesting that removal of RCD1 protein or the loss of the interaction motif in DREB2A is required for proper DREB2a function. Future experiments will show if ANAC046 and DREB2A and other transcription factors compete for interactions with RCD1, and if such interactions play a functional role during senescence (Figure 2b). In any event, the NAC transcription regulatory domains have an extreme interaction potential due to their ID, and knowledge of these domains is important for understanding of NAC interaction networks. In addition, the study of ANAC046 shows that the regions responsible for interactions of these regulatory domains can be predicted by ID analysis tools [92,101].

## 4. Local Gene Regulatory Networks of Senescence Associated NAC Genes

Multiple studies have addressed function of single NAC genes and proteins in senescence, but the knowledge about how they fit into the global GRN that controls senescence remains limited. However, recently local GRNs have emerged. Although most of the GRNs known are for Arabidopsis, a GRN has also been mapped for rice *Os*NAP. One of the most well characterized GRNs is for ORE1 [65,68], which should be a topic of a specific review and, therefore, will not be covered in this review.

### 4.1. ANAC019/ANAC055/RD26 GRN

**Figure 3:**
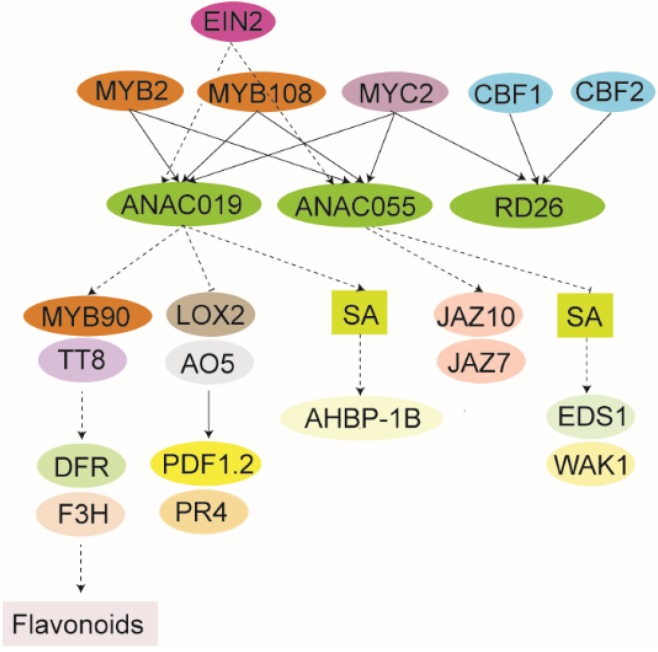
Schematic showing predicted local GRN of senescence-associated ANAC019, ANAC055, and RD726. All connections are derived from a study based on yeast one-hybrid screenings, time-course gene expression data, and mathematical modeling by Hickman *et al.* [66], except for the EIN2-ANAC019/ANAC055 and MYC2-ANAC019/ANAC055/RC26 connections which were derived from Kim *et al.* [108] and Zheng *et al.* [104], respectively. Thus, all components may not be part of the same functional GRN. The ANAC019, ANAC055, and RD26 have similar expression patterns with induction by ABA and different abiotic stress exposures [43,79,80,89]. Direct binding is shown as black lines, whereas broken lines indicate an effect which may not necessary involve a direct interaction. NAC genes/proteins are shown in green. The rest of the color coding is specific for specific compounds or proteins/genes.

The closely related NAC-a proteins, ANAC019, ANAC055, and RD26 (Figure 1), belong to a clade of stress associated NAC proteins [57,79], and their genes have similar expression patterns with induction by ABA and different abiotic stress exposures [43,79,80,89]. This is in accordance with the senescence-inducing effects of abiotic stress [11,14]. As described in Section 2.1., their senescence-associated expression patterns are also similar, both during age-dependent [27] and salt-induced senescence [43]. Based on yeast one-hybrid screenings, time-course gene expression data, and mathematical modeling Hickman *et al.* [66] proposed a senescence-associated GRN involving these three NAC proteins. Several transcription factors, including MYB2 and MYB108, capable of binding to the promoter of the *ANAC019*, *ANAC055*, and *RD26* genes, were identified. Only *ANAC019* and *ANAC055* showed a significantly reduced expression level in the T-DNA insertion mutants *myb108* and *myb2*, suggesting that MYB2 and MYB108 are not key transcriptional regulators of *RD26* during senescence (Figure 3). Microarray analysis of the T-DNA mutants *anac019* and *anac055* was also performed during developmental leaf senescence to identify down-stream components of the ANAC019 and ANAC055 GRNs. This suggested that ANAC019 is an activator of senescence through activation of flavonoid biosynthesis. Thus, the flavonoid biosynthesis pathway was down-regulated in *nac019*, as reflected by down-regulation of the biosynthetic genes *dihydroflavonol 4-reductase* (*DFR*) and *flavanone 3-hydroxylase* (*F3H*). In addition, the transcription factor genes *MYB90* and *TRANSPARENT TESTA 8* (*TT8*), both implicated in regulation of flavonoid biosynthesis [102,103], were down-regulated. They were, therefore, suggested to be direct downstream targets of ANAC019 [66].

The study by Hickman *et al.* [66] also suggested the ANAC019 and ANAC055 play different roles in regulating the antagonistic JA- and SA-signaling pathways during developmental senescence (Figure 3). ANAC019 may enhance SA-signaling and repress JA-signaling, and ANAC055 may be required for normal JA-signaling. *anac019* showed increased expression of the JA-biosynthesis genes *LIPOXYGENASE 2* (*LOX2*) and *ALLENE OXIDE SYNTHASE* (*AOS*) and the JA-responsive genes *PATHOGENESIS-RELATED-44* (*PR4*) and *PLANT DEFENSIN 1.2* (*PDF1.2*). For *anac055*, reduced expression of the JA-signaling genes *JASMONATE ZIM-DOMAIN 7 (JAZ7)* and *JAZ10* was observed. In a previous study by Bu *et al.* [85] ANAC019 and ANAC055 were suggested to have similar roles in regulating JA-induced expression of defense genes. *anac019* *anac055* double mutant plants showed attenuated JA-induced *VSP1* and *LOX2* gene expression, whereas transgenic plants overexpressing the two NAC genes showed the opposite effects. That JA-induced expression of the two NAC genes depends on the function of *CORONATINE-INSENSITIVE1* (*COI1*) and *AtMYC2*, together with the finding that overexpression of *ANAC019* partially rescued the JA-related phenotype of the *atmyc2-2* mutant [85], led to a hypothesis that the two NAC proteins act downstream of *At*MYC2 to regulate JA-signaled defense responses. In support of this, it was recently shown by chromatin immunoprecipitation (ChIP) that *ANAC019*, *ANAC055*, and *RD26* are direct *in vivo* targets of *At*MYC2 [104].

ANAC019 and ANAC055 also seem to have opposite roles in SA-signaling related to senescence. Based on decreased expression of *AHBP-1B*, encoding a transcriptional repressor implicated in SA-signaling [105], the SA pathway may be down-regulated in the *anac019* mutant, whereas up-regulation of the SA pathway in the *anac055* mutant was suggested by enhanced expression of e.g., *CELL WALL-ASSOCIATED KINASE 1* (*WAK1*), expressed in response to SA [106], and *ENHANCED DISEASE SUSCEPTIBILITY 1* (*EDS1*), which is involved in SA-mediated signaling in defense [107].

The senescence-associated GRN of ANAC019 and ANAC055 may also involve components of the EIN2 senescence-regulating GRN [59,108], described further in Section 4.2. Thus, *ANAC019* and *ANAC055* were identified as candidate downstream components of EIN2 in a comparative gene expression analysis [108]. However, there are contradicting reports as to whether *ANAC019* and *ANAC055* are direct targets of the transcription factor EIN3, a downstream component of EIN2 in the ethylene signaling cascade [109]. Whereas Kim *et al.* [108] did not show direct binding of EIN3 to the promoters of *ANAC019* and *ANAC055*, Chang *et al.* [110] showed that EIN3 bound to the promoters of four NAC genes, *ANAC019*, *ANAC055*, *SHG*, and *ORS1*, after ethylene treatment in *Arabidopsis* seedlings. However, the expression of these NAC genes was not altered in the mature leaves of the *ein3 ethylene insensitive 3-like 1* (*eil1*) double mutant [108] suggesting that they are regulated primarily by other transcription factors that act downstream of EIN2 in a mature leaf (Figure 3).

Four AP2 transcription factors, C-REPEAT/DEHYDRATION RESPONSIVE ELEMENT BINDING FACTOR (CBF1), CBF2, CBF3, and CBF4 bound to only the *RD26* promoter, and modelling suggested that CBF1 and CBF2 are important for the senescence response [66]. *CBF2* and *CBF3* have also been implicated in senescence, since overexpression of these genes resulted in delayed leaf senescence in Arabidopsis [111]. Thus, the CBF transcription factors may regulate leaf senescence via *RD26* as a target gene. Together the different studies demonstrate the complexity and intersections of the senescence-associated GRNs of ANAC019, ANAC055, and RD26, which may play important regulatory roles in leaf senescence.

### 4.2. AtNAP GRN

**Figure 4:**
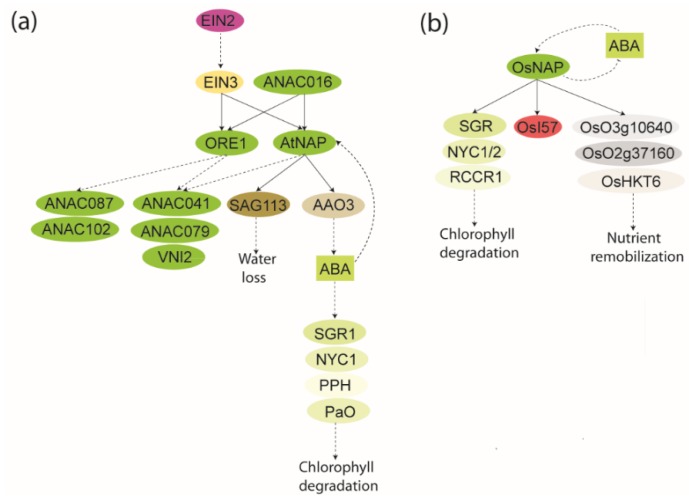
Schematic showing local GRN of senescence associated NAP. (**a**) GRN of *At*NAP. The AtNAP-SAG113 connection is derived from Zhang *et al.* [86], the EIN2-EIN3-ORE1/AtNAP/ANAC087/ANAC102/ANAC041/ANAC079/VNI2 connections are derived from Kim *et al.* [108], the ANAC016-*At*NAP connection is derived from Kim *et al.* [63], and the AtNAP-AAO3-ABA-SGR1/NYC1/PPH/PaO connection is derived from Yang *et al.* [87]. Thus, all components may not be part of the same functional GRN (**b**). GRN of *Os*NAP. Genes downstream of *Os*NAP are related to chlorophyll degradation and nutrient transport. The connections are derived from Liang *et al.* [28]. Direct binding is shown by black lines, whereas broken lines indicate an effect which may not necessarily involve a direct interaction. NAC genes/proteins are shown in green. The rest of the color coding is specific for specific compounds or proteins/genes.

The significant role of *At*NAP, also a subfamily NAC-a NAC proteins, in senescence became clear about a decade ago. It was shown that a T-DNA insertion knockout of *AtNAP* displayed delayed leaf senescence characterized by delayed loss of chlorophyll content, and that overexpression of *At*NAP in young leaves caused precocious senescence. Together this suggested that *At*NAP is a positive regulator of senescence [58]. *SAG113* is a direct target gene of *At*NAP, and its expression depends primarily on *At*NAP (Figure 4a). In accordance with this, overexpression of SAG113 restored the delayed leaf senescence phenotype of the *atnap* mutant to wildtype [86]. *SAG113* is an ABA and senescence up-regulated protein phosphatase 2C and is localized in the *cis*-Golgi apparatus. As a negative regulator of ABA-signaling, SAG113 specifically suppressed stomata closure. This resulted in rapid water loss in senescing leaves and thereby senescence and desiccation [112]. Thus, ABA, *At*NAP, and SAG113 may together control stomatal movement and water loss during leaf senescence.

A pathway involving *EIN2*, *ORE1*, and *miR164* in Arabidopsis has been shown to regulate age-dependent leaf senescence and cell death [59]. EIN2 is required for ethylene responses [113], and as mentioned in Section 4.1 the transcription factor EIN3 is a downstream component of EIN2 in the ethylene signaling cascade [109]. The six NAC genes, *ORE1*, *ANAC019*, *AtNAP*, *SHG*, *ANAC055*, and *ORS1* were identified as candidate downstream components of EIN2 [108]. EIN3 directly bound the *ORE1* and *AtNAP* promoters (Figure 4a) and transcriptionally activated both genes. Both *ORE1* and *AtNAP* were required by EIN3 to induce leaf senescence, suggesting that EIN3 positively regulates leaf senescence by activating the *ORE1* and *AtNAP* genes. ORE1 and *At*NAP were, therefore, analyzed for additive and differential functions in leaf senescence. The *ore1 atnap* double mutant exhibited stronger senescence phenotypes than either of the single mutants suggestive of additive regulation of leaf senescence by the two NAC genes. Since NAC genes are themselves likely NAC target genes in senescence [27,45,65], the transcript level of seven senescence-associated NAC genes were evaluated in wild-type and *ore1*/*atnap* leaves. This revealed some NAC genes, *ANAC041*, *ANAC079*, and *VNI2*, regulated by both ORE1 and *At*NAP, and two NAC genes, *ANAC087* and *ANAC102*, regulated by only ORE1 suggestive of both distinct and overlapping signaling pathways for ORE1 and *At*NAP in senescence [108]. Identification of direct downstream target genes of ORE1 and *AtNAP* is essential to provide new insight into how the GRN involving EIN2-mediated senescence signaling regulates the leaf senescence process.

Recently, the functional role of *At*NAP in chlorophyll degradation was dissected [87]. In excised leaves, *AtNAP* expression was induced by ABA, and the gene was co-expressed with several ABA metabolism and signaling genes. In addition, the transcript levels of the ABA biosynthetic genes *9-CIS-EPOXYCAROTENOID DIOXYGENASE 2*, *ABA DEFICIENT 3*, and *AAO3* were low in dark-treated *nap* leaves, and the ABA level was extremely low in *nap* leaves during extended darkness. *At*NAP bound the *AAO3* promoter and activated its transcription, and overexpression of *AAO3* in *nap* leaves suppressed the stay-green phenotype of *nap* under extended darkness. It was also shown that ABA up-regulated the chlorophyll degradation genes, *STAY-GREEN1* (*SGR1*), *NON-YELLOW COLORING1* (*NYC1*), *PHEOPHYTINASE* (*PPH*), and *PHEIDE a OXYGENASE* (*PaO*) suppressed the stay-green phenotype of *nap*. Based on these results a model was proposed according to which *At*NAP promotes chlorophyll degradation by enhancing transcription of *AAO3*, which results in increased levels of ABA. This in turn, via unknown transcription factors, leads to increased expression of chlorophyll degradation genes and eventually chlorophyll degradation [87] (Figure 4a).

ANAC016, belonging to subfamily NAC-b containing several membrane-anchored NAC proteins, recently caught attention in relation to senescence because of its expression pattern [63]. The *anac016* mutant remained green under senescence-inducing conditions, and overexpression of *ANAC016* accelerated leaf yellowing during senescence. Under dark-induced senescence several SAGs including *JUB1*, *AtNAP*, *ORE1*, *ORS1*, and *VNI1* were down-regulated in *nac016* mutants and up-regulated in NAC016 overexpressing plants, and ANAC016 bound both the *AtNAP* and *ORS1* promoters. *anac016* mutants also exhibited delayed senescence under salt and H_2_O_2_ stresses. That *AtNAP* may be a direct target gene of ANAC016 is supported by the observation that the *atnap* and *anac016* mutants have similar stay-green phenotypes under salt and oxidative stress conditions and during natural and dark-induced senescence. Therefore, ANAC016 likely adds another link, involving salt and oxidative stress, to the *At*NAP GRN (Figure 4a).

### 4.3. OsNAP GRN

*Os*NAP also acts as a key component linking ABA-signaling and leaf senescence, as shown in a recent study by Liang *et al.* [28]. To identify genes controlling leaf senescence in rice, the authors screened a T-DNA population for gain-of-function mutants and identified *prematurely senile 1* (*ps1-D*). *PS1* encodes the *At*NAP ortholog, *Os*NAP (*ONAC058*), which complemented the *atnap* mutant [58,114]. *OsNAP* was induced during senescence and by ABA [28,115], and the ABA content was reduced in *ps1-D* mutants [28]. RNAi transgenic lines, repressing *OsNAP* expression, displayed delayed leaf senescence and decreased levels of several SAGs, including chlorophyll degradation-related genes. ChIP assays revealed that *Os*NAP bound the promoter of several of these and also activated transcription from *SGR*, *NYC1*, *NYC3*, *RED CHLOROPHYLL CATABOLITE REDUCTASE*(*RCCR1*), and *Os*I57*. Os*NAP also directly targeted genes related to nutrient transport and bound the promoters of genes encoding high-affinity potassium transporter 6 (*Os*HKT6), heavy metal transport/detoxification protein (*Os02g37160*) and calcium transporting ATPase (*Os03g10640*). This suggests that *OsNAP* is an ideal marker of senescence onset in rice. Thus, leaf senescence resulted in accumulation of *OsNAP*, which regulated genes related to chlorophyll degradation and nutrient transport and controlling senescence in an ABA-dependent manner. ABA up-regulated *OsNAP* expression, whereas *Os*NAP down-regulated ABA biosynthesis via a feedback mechanism (Figure 4b). *Os*NAP links ABA and leaf senescence by regulating ABA-biosynthesis and targeting SAGs in rice. Importantly, reduced *Os*NAP expression resulted in an extended grain filling period and increased grain yield [28]. Therefore, fine-tuning *Os*NAP expression may be used to improve rice yield in the future.

So far, most molecular studies of senescence have been performed in Arabidopsis because of the many advantages associated with a model organism for such studies. However, functional divergence of orthologous NAC transcription factors [116] may hamper the translation of results from this model plant to crop plants. *At*NAP and *Os*NAP show both similarities and differences in their manner of connecting ABA and leaf senescence. Although *Os*NAP was also suggested to function as a transcriptional activator that plays a role in ABA-dependent mediation of stress responses, the protein did not activate transcription from the promoters of *OsPP2C09* and *OsPP2C68*, which are homologs of *SAG113* [115]. Therefore, *Os*NAP is likely to mediate its ABA-associated function in a manner different from the function of *At*NAP in the ABA-*At*NAP-SAG113 chain during leaf senescence in *Arabidopsis* [58]. However, both *At*NAP and *Os*NAP function through ABA-and SAG-dependent pathways to mediate chlorophyll degradation [28,87], a hallmark of senescence, although *Os*NAP inhibited and *At*NAP promoted ABA-biosynthesis, respectively. In addition to accumulating during senescence and in response to ABA, *OsNAP* transcripts accumulated in response to JA-treatment [114]. JA levels were up-regulated in *OsNAP* overexpressing transgenic plants and down-regulated in RNAi lines compared to wildtype plants. In addition, *LOX2* and *ALLENE OXIDE CYCLASE* from the JA-biosynthetic pathway were induced in *OsNAP* overexpressing plants compared to wildtype plants, whereas *OsNAP* RNAi lines showed relatively low levels of these JA-biosynthetic genes. This suggests that the function of *Os*NAP as a positive regulator may also involve the JA-pathway [114], a complexity that has so far not been investigated for *At*NAP. Further studies will reveal if there is a synergistic effect between ABA and JA, as suggested for the *ANAC019*, *ANAC055* and *RD26* genes (Section 2.1) [66], in senescence.

### 4.4. ATAF1 GRN

**Figure 5:**
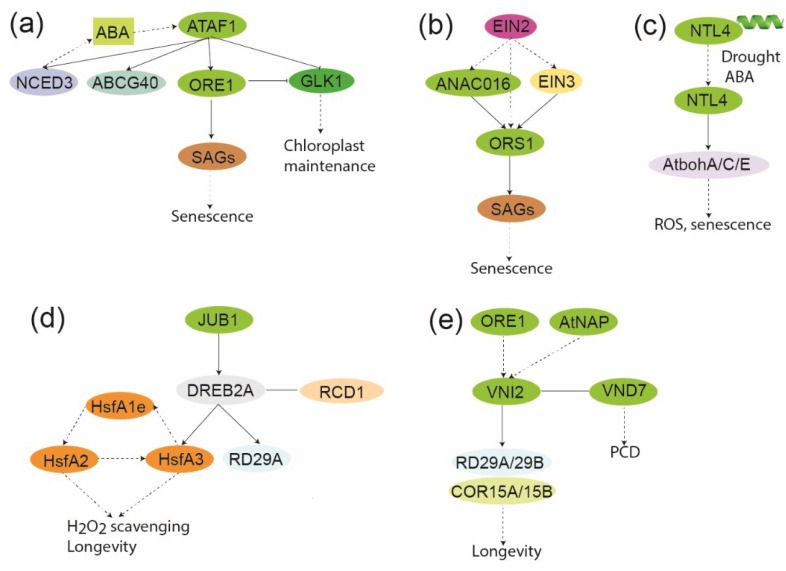
Schematic showing local GRN of senescence associated NAC genes (**a**) GRN of ATAF1. The ATAF1-NCED3/ABCG40/ORE1/GLK1 connection is derived from Garapati *et al* [64]. The interaction between ORE1 and GLK1, inhibiting GLK1 is derived from Rauf *et al.* [68]. The balance between ORE and GLK1 determines the status of senescence. The connection between ATAF1 and SnRK1.1 was derived from Kleinow *et al.* [121]. *ATAF1* expression is regulated by both ABA and H_2_O_2_ through unknown transcription factors [64,80]; (**b**) GRN of ORS1. The ORS1-SAG connection is derived from Balazadeh *et al.* 2011 [60], the ANAC016-ORS1 connection is derived from Kim *et al.* [63], the EIN2-ORS1 connections are derived from Kim *et al.* [108], and the EIN3-ORS1 connection is derived from Chang *et al.* [110]. Thus, all components may not be part of the same functional GRN. *ORS1* expression is induced by H_2_O_2_ through unknown transcription factors (**c**) GRN of NTL4. Drought and ABA mediate proteolytic processing of NTL4. The NTL4-AtbohA/C/E connections are derived from Lee *et al.* [90]. *NTL4* expression is induced by ABA, drought, and heat. The figure was inspired by a recent review [123]; (**d**) GRN of JUB1. The JUB1-DREB2A connection is derived from Wu *et al.* [61], whereas the additional connections are from earlier work [99,124,125,126]. Thus, all components may not be part of the same functional GRN. *JUB1* expression is induced by H_2_O_2_ through unknown transcription factors. The figure was inspired by a recent review [123]; (**e**) GRN of VNI2. The VNI2-COR15/COR15B/RD29A/RD29B connection is derived from Yang *et al.* [62], the VNI2-VND7 interaction is derived from Yamaguchi *et al.* [127], and the ORE1/*At*NAP connection is derived from Kim *et al.* [108]. Thus, all components may not be part of the same functional GRN. The expression of *VNI2* is induced by salt in an ABA-dependent manner. Direct binding is shown by black lines, whereas broken lines indicate an effect which may not necessarily involve a direct interaction. NAC gene/proteins are shown in green. The rest of the color coding is specific for specific compounds or proteins/genes.

Recently another subfamily NAC-a member, ATAF1, was also shown to promote senescence by coupling ABA hormone signaling with down-regulation of photosynthesis-associated genes and up-regulation of SAGs [64] (Figure 5a). As for other subfamily NAC-a members, the expression of *ATAF1* was induced by different abiotic stresses [79,117] including H_2_O_2_ [118] and ABA [64,80,117]. Garapati *et al.* [64] showed that overexpression of *ATAF1* promoted both developmental and dark- and ABA-induced senescence, while *ataf1* knockout plants displayed delayed senescence. The Myb transcription factor *GLK1* and *ORE1* were among the genes which were differentially expressed upon changes in the *ATAF1* level, and ATAF1 directly bound the promoters of these two genes (Table 1). Hereby, ATAF1 up-regulated the expression of the *ORE1* gene, whereas it down-regulated the expression of the *GLK1* gene. In contrast to ORE1, which is a positive regulator of senescence [59,65], GLK1 together with GLK2 function in chloroplast development and maintenance [119,120], and overexpression of *GLK1* and *GLK2* resulted in delayed senescence [68]. ORE1 has previously been shown to promote senescence by antagonizing GLK-mediated transcription through direct interaction with the GLK proteins [68]. Thus, ATAF1 regulates senescence by balancing the ORE1 and GLK1 levels and activity thereby regulating photosynthesis-related genes. The function of ATAF1 in senescence also involved both ABA and H_2_O_2_, which up-regulate *ATAF1* and *ORE1* and down-regulate *GLK1*, suggesting that stress-dependent regulation of *ORE1* and *GLK1* is also mediated by ATAF1. Both the ABA-biosynthesis gene, *NCED3*, and the ABA-transporter, *ABCG40*, were direct targets of and regulated by ATAF1 [64]. ATAF1 had previously been shown to bind the *NCED3* promoter (Table 1), and in accordance with this *ATAF1* overexpressing plants had significantly increased ABA levels compared to wildtype plants and *ataf1* mutants [88]. Thus, ATAF1 may regulate ABA biosynthesis in a positive feed-back loop by binding to and activating the expression of *NCED3*. Plants overexpressing *ATAF1* display stunted growth and delayed flowering, which is also the case for ABA-hypersensitive plants overexpressing the ATAF1 interaction partner kinase *SNF1-RELATED SERINE/THREONINE PROTEIN KINASE1* (*SnRK1.1*) [121,122]. This interaction also involved the intrinsically disordered transcriptional regulatory domain of ATAF1, again demonstrating the interaction potential of this NAC region [121,123]. Further studies will show if this interaction is also relevant to senescence. In conclusion, ATAF1 links ABA- and H_2_O_2_-signaling with regulation of photosynthesis-associated genes and SAGs in the senescence process [64].

### 4.5. ORS1

ORS1, a subfamily NAC-d member, is also a positive regulator of senescence, with overexpression and knockout of the *ORS1* gene promoting and delaying leaf senescence, respectively [60]. Transcriptome analysis revealed that 76% and 67% of the ORS1 regulated genes were induced by long term salinity stress and H_2_O_2_ treatment, respectively, and *ORS1* itself was also strongly induced by H_2_O_2_ [60]. As mentioned in Section 4.2, ANAC016, the gene of which is also up-regulated by salt and H_2_O_2_ stress, binds the *ORS1* promoter [63]. Thus, ORS1 induces expression of SAGs through a GRN that may involve salt- and H_2_O_2_-dependent signaling pathways [60] and has ANAC016 as a direct regulator of *ORS1* (Figure 5b). ORS1 may also be part of the EIN2/EIN3 GRN, since the *ORS1* gene was recently identified as a down-stream component of EIN2 [108]. However, since the expression kinetics of *ORS1* during leaf ageing was similar in wildtype plants and the *ein2* mutants, *ORS1* expression might be controlled by both EIN2 independent and EIN2 dependent senescence signals. Chang *et al.* [110] showed that EIN3 bound to the promoters of four NAC genes, including *ORS1*, after ethylene treatment in *Arabidopsis* seedlings. However, the expression of these NAC genes was not altered in the mature leaves of the *ein3 eil1* double mutant [108] suggesting that ORS1 is regulated primarily by other transcription factors that act downstream of EIN2 in a mature leaf.

### 4.6. NTL4

The ABA, drought, and heat inducible *NTL4* gene encodes a subfamily NAC-b membrane-anchored NAC protein which plays an important role in drought-induced senescence by promoting ROS production [90]. In addition to *NTL4* gene up-regulation, drought stress mediated ABA-dependent membrane release of plasma membrane bound NTL4 by proteolysis (Figure 5c). NTL4 directly up-regulated the *Atrboh A*, *C*, and *E* genes encoding NADPH oxidases that are involved in ROS production thereby triggering PCD during drought-induced leaf senescence. Accordingly, leaf senescence was accelerated in transgenic plants overexpressing proteolytically processed *NTL4* under drought conditions. In addition, these transgenic plants were hypersensitive to drought and ROS accumulated in the leaves. In contrast, the *ntl4* mutants were resistant to drought stress. Together these observations indicate that NTL4-regulated ROS production contributes to the process of drought-induced leaf senescence in Arabidopsis [90]. More recent data suggest that NTL4 is also involved in ROS-induced PCD under heat stress conditions [128]. In conclusion, membrane-anchored NTL4 is a node connecting abiotic stress responses with senescence.

### 4.7. JUB1

JUB1 is the only subfamily NAC-e member which has been characterized in relation to senescence. JUB1 is a negative regulator of senescence and increased longevity in Arabidopsis [61]. This H_2_O_2_-inducible NAC gene most likely mediates its effects through a GRN (Figure 5d) that controls the level of intracellular H_2_O_2_. *JUB1* overexpression resulted in delayed senescence, decreased H_2_O_2_ levels and enhanced tolerance to abiotic stresses such as heat and salt, whereas the *jub1-1* mutant had the opposite effect. JUB1 increased the expression of several H_2_O_2_ responsive genes, and H_2_O_2_ treatment further stimulated this increase. Thus, as mentioned in Section 3.1., JUB1 directly activated the AP2 transcription factor gene *DREB2A*, which is important in abiotic stress responses [124]. DREB2A in turn directly targets the *RESPONSIVE TO DESSICATION 29A* (*RD29A*) [124] and *HsfA3* [125] genes. Furthermore, Hsf3 has been suggested to be part of a positive feedback loop together with HsfA1e and HsfA2 [126]. HsfA2 and HsfA3 in turn regulate the expression of genes encoding HEAT SHOCK PROTEINS (HSPs) and H_2_O_2_ scavenging enzymes [129,130,131,132]. Based on the study by Wu *et al.* [61], JUB1 was proposed to mediate lowering of the intracellular H_2_O_2_ level through the HsfA3-associated feedback loop, thereby resulting in prolonged longevity and increased stress tolerance (Figure 5d). In accordance with the enhancement of abiotic stress tolerance, metabolite profiling using gas chromatography coupled to mass spectrometry showed increased levels of the amino acid proline and the disaccharide trehalose upon overexpression of *JUB1* [61,133,134]. As mentioned in Section 3.2, a splice variant of DREB2A, which lacks the RCD1 interacting motif, accumulated during heat stress and senescence [99], suggesting that removal of RCD1 protein or the loss of the interaction motif in DREB2A appears to be required for proper DREB2a function. Further studies will show if the DREB2A interaction partner and regulator, RCD1 [99], functionally contributes to the senescence-associated GRN shown in Figure 5d.

### 4.8. VNI2

VNI2, of subfamily NAC-f, is also a negative regulator of senescence which regulates senescence through integration of ABA signaling [62]. In addition it regulates xylem vessel specification [127]. The expression of *VNI2* was induced during senescence and by salt, and the expression was dependent on ABA. Overexpression of *VNI2* enhanced resistance to salt stress and prolonged leaf longevity, which is also the case for the direct target genes, senescence-induced *COR15A*, *COR15B*, *RD29A*, and *RD29B* (Figure 5e) [62], which are marker genes for abiotic stress responses. VNI2 interacted with another NAC protein, VASCULAR-RELATED NAC-DOMAIN7 (VND7), which is associated with PCD [127]. Together this indicates that the VNI2-centered GRN of ABA-regulated abiotic stress responses in senescence may, as mentioned in Section 4.2, also include ORE1 and *At*NAP [108]. In this case, the positive regulators of senescence, ORE1 and *At*NAP, activate *VNI2*, which is a negative regulator of leaf senescence [62], thereby fine-tuning the process of senescence.

### 4.9. NAC in GRNs

Emerging local NAC-centered GRNs reveal glimpses of the complexity expected for such networks. In this review, information from several independent studies is included in the GRNs, when available. Although not all of the connections may be integrated and physiologically relevant, this reveals new connections of putative importance. All of the central NAC transcription factor genes shown in the GRNs (Figure 2, Figure 3 and Figure 4) are differentially regulated during senescence [27,28,43,64]. In addition, most of these genes are also regulated by hormones, with ABA being especially dominant. This phytohormone contributes directly to the molecular mechanism of the *At*NAP-, *Os*NAP-, ATAF1-, NTL4-, and VNI2-centered GRNs. ANAC019, ANAC055, and RD26 are also regulated by ABA, although its role in the molecular mechanisms of these NAC proteins in senescence remains elusive. All of the GRNs described have central NAC genes, which are also induced by various abiotic stress responses, which also affect the senescence process. Thus, NAC transcription factors play a significant role in mediating the widely known role of abiotic stress factors in senescence [11,14]. Although this is striking, it is not unexpected based on the global expression patterns of the NAC gene family revealing extensive gene regulation by exposure to abiotic stresses [57,79]. Thus, NAC transcription factors are central to ABA- and stress-dependent signaling in senescence which also involves SAGs and in the case of *At*NAP, *Os*NAP, and ATAF1 components of photosynthesis cascades. Together the NAC GRNs illustrate some of the most well-established characteristics of senescence such as hormonal and stress regulation and chlorophyll degradation. The NAC GRNs also demonstrate extensive cross-talk between NAC transcription factors in senescence, with both direct and indirect regulation of NAC genes by NAC proteins. Curiously, the activity of the negative senescence regulator VNI2 may be fine-tuned by the positive senescence regulators *At*NAP and ORE1. Whereas most of the senescence-associated NAC genes belong to the large and well-characterized NAC subfamilies, NAC-a, NAC-b, NAC-d [55,56,57], the important negative senescence regulators VNI2 and JUB1 belong to the small less characterized subfamilies NAC-e and NAC-f, respectively, suggesting that many more NAC genes with significant and maybe unexpected roles in senescence are to be revealed.

## 5. Crop NAC Genes with an Effect on Grain Yield and Quality

Senescence has a significant impact on crop productivity, and manipulation of the senescence process could result in higher yield. In 1971, Lawes and Treharne [135] reported a direct correlation between yield and flag leaf area duration. It has also been shown that plants with stay-green phenotypes have the potential for higher productivity due to a longer period of active photosynthesis. However, these plants also exhibit an inefficient nitrogen remobilization and therefore a low harvest index [47,136,137]. On the other hand, accelerated senescence causes efficient nitrogen remobilization and a high grain protein content, but also a lower total grain yield [138]. Breaking this negative correlation between grain yield and grain protein concentration has caused difficulty in cereal breeding programs [136]. However, recent studies suggest that NAC genes are promising targets for breeding for increased grain yield and quality.

### 5.1. NAM-B1

About a decade ago a direct link was demonstrated between senescence and nutrient distribution from studies of *Grain protein content (Gpc)-B1*, a quantitative trait loci (QTL), from wild emmer wheat [67]. Previously, *Gpc-B1* had been shown to affect GPC in tetraploid and hexaploid wheat, to influence the remobilization of nitrogen from flag leaves to ears during grain filling [139], and to accelerate senescence in flag leaves [140]. The gene for this trait, *NAM-B1*, encodes a NAC protein, which is most closely related to the Arabidopsis NAC proteins ANAC025, NAM/NARS2 (ANAC018) and NARS1/NAC2 (ANAC056) belonging to the same subfamily, NAC-a, as *At*NAP. Whereas wild emmer has a functional copy of the gene, most modern wheat varieties have a non-functional copy of the gene [67,141]. In addition to *NAM-B1*, wheat contains two other homeologous *NAM1* genes, and the paralogous gene *Gpc2* (*NAM2*) that shows 91% sequence similarity with *Gpc*1 [67]. The *NAM* genes showed increased expression during senescence of flag leaves, and silencing of the genes with RNAi resulted in transgenic plants with delayed senescence and more than 30% reduction of grain zinc, iron, and protein content associated with increased residual nitrogen, zinc, and iron content in the flag leaf. So, the *NAM* genes mediate nutrient redistribution to the developing grain during leaf senescence [67]. That the *Gpc* genes play a significant role during early senescence was further suggested from comparative transcriptome analysis of *Gpc-RNAi* lines and control lines at an early stage of senescence. The approximately seven hundred differentially expressed genes included transporters, hormone-regulated genes and transcription factors, many of which were also up-regulated during senescence [142]. A recent study focused on the *Gpc-B1* homologs, *Gpc-A1* and *Gpc-D1*, by isolation and characterization of knockout mutants for these genes [143]. The single and double mutants were grown under field conditions at four locations and characterized. All knockout mutants showed a reduction in the rate of total N accumulation in the grains during the second half of the grain filling period compared to wildtype plants, whereas grain weight at maturity was unaffected by the mutations. The *Gpc1* mutants also exhibited delays in senescence and in grain Zn and Fe content. In contrast, knockout of *Gpc2* did not affect these traits, stressing the role of *Gpc1* as a key regulator of nutrient remobilization during early senescence [144].

Because of the critical role played by the *Gpc* genes in leaf senescence and nutrient redistribution to the developing grain, other crop plants have been analyzed for orthologous genes. In rice, *Os07g37920* is the closest homolog of *Gpc-B1* and *Gpc-2B*. Despite of this, RNAi and overexpression of *Os07g37920* did not result in differences in senescence. In contrast, the gene was shown to be important for another development in rice. So, this demonstrates divergent functions of orthologous *Gpc NAC* genes in wheat and rice, and precludes the use of rice as a model for studies of *Gpc* in senescence and nutrient remobilization [116].

In barley, two genes, *HvNAM-1* and *HvNAM-2*, encoding homologs of the *Gpc-B1* were identified and mapped to chromosomes 6H and 2H, respectively [67]. Barley chromosome arm 6HS was shown to be associated with a QTL for GPC using a barley recombinant inbred line population developed from a cross between “Karl”, a low grain protein six-rowed variety and “Lewis”, a high grain protein two-rowed variety [138,145,146]. A high degree of colinearity between the barley 6HS and wheat 6BS GPC QTLs revealed that *HvNAM-1* is the ortholog of *Gpc-B1* [147]. Allelic diversity of *HvNAM-1* among wild and cultivated barley explained variation in GPC, suggesting that the biological function of *HvNAM-1* is similar to that of *Gpc-B1* [148,149]. Furthermore, the near-isogenic barley line “10_11” derived from a backcross of high-GPC locus from “Lewis” to “Karl” showed higher GPC and accelerated senescence compared to “Karl”, and a phenotype similar to the *Gpc-B1* wild emmer introgression line [48,150,151,152]. In addition, the barley GPC locus was shown to influence pre-anthesis plant development and leaf senescence, but only after floral transition at the shoot apical meristem occurred [153,154]. The high-GPC near-isogenic line “10_11” developed faster and flowered five days earlier than the low-GPC line [153]. Moreover, transcriptome analyses identified several genes which are highly up-regulated in senescing “10_11” as compared with “Karl” leaves, and which therefore could be involved in senescence regulation [48]. These included several leucine-rich repeat receptor protein kinase genes and a gene coding for a barley glycine-rich RNA binding protein 1 (HvGR-RBP1) with high sequence homology to *Arabidopsis* glycine-rich RNA binding protein 7 (AtGRP7) [154], which has previously been implicated in the promotion of flowering [155]. Recently, *HvGR-RBP1* was localized to barley chromosome 6, supporting the interpretation that it may be linked to *HvNAM-1* [152]. These results suggest that NAM transcription factors are involved in senescence and nutrient remobilization not only in wheat, but also in other cereals.

### 5.2. TaNAC-S

The wheat NAC gene, *TaNAC-S*, closely related to Arabidopsis and rice NAC1 of subfamily NAC-a, was identified in a recent transcriptomics study analyzing for genes with expression profiles showing positive correlation with leaf nitrogen concentration [156]. The expression of *TaNAC-S* decreased during leaf senescence, and overexpression of *TaNAC-S* in transgenic wheat resulted in a stay-green phenotype, indicating that *Ta*NAC-S is a negative regulator of senescence. Furthermore, these stay-green *TaNAC-S* overexpressing plants showed higher grain nitrogen concentration than wildtype plants at similar grain yields. Thus, *TaNAC-S* may be a target for future wheat improvement strategies.

### 5.3. OsNAP

As mentioned in Section 4.3, Liang *et al.* [28] showed that *OsNAP* knockout plants had a marked delay in senescence characterized by a significantly slower decrease in photosynthetic capacity and an extended grain-filling period compared with wildtype plants. This resulted in a 6.3% and 10.3% increase in the grain yield of two independent RNAi lines. In this study, no data were provided for the grain protein content of the *OsNAP* knockout plants compared to wildtype plants. In another study, Chen *et al.* [115] focused on the relationship of *Os*NAP to abiotic stress responses and ABA. The authors demonstrated that rice lines overexpressing *OsNAP* showed a lower rate of water loss and increased tolerance to high salinity, drought and low temperature at the vegetative stage compared to wildtype plants. Thus, fine-tuning of *OsNAP* expression is also promising as a strategy for improving rice yield in the future.

### 5.4. GhNAP

Recently NAP was also analyzed in cotton [157], for which premature leaf senescence has become one of the main factors restricting yield and quality [158]. *GhNAP* responded rapidly to senescence signals, and, therefore, represents a useful senescence marker in cotton. Like *OsNAP* [58,114], *GhNAP* complemented the senescence phenotype of the *anap* null mutant [157]. In addition, overexpression of *GhNAP* resulted in early senescence in *Arabidopsis*, whereas down-regulation of *GhNAP* resulted in delayed senescence in cotton. Down-regulation of *GhNAP* also resulted in increased cotton yield, and fibre quality, especially length, was changed. The function of *Gh*NAP was also related to ABA. Thus, *GhNAP* was highly induced by ABA-treatment, and down-regulation of *GhNAP* resulted in decreased ABA levels and reduced expression of ABA-responsive genes, including *GhSAG113.* However, *Gh*NAP failed to bind directly to the *SAG113* promoter suggesting that ABA-dependent regulation of senescence by *GhNAP* is different from the ABA-*At*NAP-SAG113 regulatory chain in *Arabidopsis* during leaf senescence [86,157]. In conclusion, *Gh*NAP also represents a candidate gene for future plant breeding.

### 5.5. HvSNAC1

The examples above clearly demonstrate how NAC genes play central roles in senescence processes involving abiotic stress responses. However, NAC genes also respond to various biotic stresses (see e.g., [56,159,160]). Furthermore, several NAC genes, *ANAC019*, *ANAC032*, *SHYG*, *ANAC055*, and *ORE1* were up-regulated both by infection with the necrotrophic fungus *Botrytis cinera* and by dark-induced senescence, and the expression of these genes was also affected by the nitrogen supply [41,161,162]. Recently, barley stress-responsive NAC1 (*Hv*SNAC1), previously named *Hv*NAC003 [50] and in Arabidopsis most closely related to ATAF2 of subfamily NAC-a (Figure 1), was suggested to be implicated in both senescence and resistance to Ramularia leaf spot (RLS) [163]. RLS has recently become an important disease of barley across Europe [164] and is caused by the ascomycete fungus *Ramularia collo-cygni*. The rice ortholog of *Hv*SNAC1, SNAC1, plays a role in drought and salt tolerance and enhanced the expression of stress-responsive genes [165] and regulated genes that control stomatal closure and ROS homeostasis [166]. In addition, overexpression of both *SNAC1* and *HvSNAC1* enhanced drought tolerance in transgenic plants compared to wildtype plants [163,165]. Interestingly, overexpression of *HvSNAC1* in barley cv. Golden Promise also resulted in an increase in resistance towards RLS. Although *HvSNAC1* overexpression affected the development of RLS, it had no effect on four other diseases, suggesting that the effect on RLS was specific. Overexpression of *HvSNAC1* also delayed dark-induced leaf senescence in the transgenic lines. Studies of the link between RLS, senescence and stress responses may provide insights both into why RLS has recently emerged as an important disease of barley and into the association of biotic stress resistance and senescence [163].

### 5.6. PopNAC154

Genome-wide analysis revealed 153 NAC genes in Populus [167], some of which are now being characterized. *PopNAC154*, which is one of two Populus co-orthologs of *SECONDARY WALL-ASSOCIATED NAC DOMAIN PROTEIN 2* (*SND2*; *ANAC073*) [168] of subfamily NAC-g (Figure 1) was expressed in both developing secondary xylem and phloem fibers, and overexpression of this NAC gene resulted in green-house grown trees with reduced height and an increased relative proportion of bark *versus* xylem compared to control plants [169]. Metabolic profiling using a non-targeted approach based on a liquid chromatography mass spectrometry platform was used to assess the effect of *PopNAC154* overexpression in field grown poplar stem wood [170]. This revealed changed phenolic glycoside, oligolignol, and sucrose levels. Interestingly, increased levels of arginine were also detected in *PopNAC154* overexpressing tissues. Leaf senescence and dormancy in Populus have been shown to correlate with increasing levels of arginine, representing a nitrogen storage compound, in stem wood [171]. Together these data indicate that *Pop*NAC154 plays a role in the senescence/dormancy processes. A direct correlation between arginine and field phenotype such as height and composite leaf angle was also observed, suggesting that *PopNAC154* may be a target for future genetic manipulation of poplar growth and development [170].

### 5.7. Crop NAC Genes for Breeding

During senescence, genetically-programmed and developmentally-controlled catabolic activities convert cellular material into exportable nutrients that are remobilized from the leaves to the developing grains [116]. Therefore, senescence and nutrient remobilization are closely connected processes, and future improvement of agronomic traits such as yield and grain quality require a better understanding of GRNs controlling both processes [152]. It is now becoming clear that several members of the NAC transcription factor family have a crucial impact on regulation of senescence and the nutrient remobilization process in cereals. The demonstration by Uauy *et al.* [67] that *Gpc-B1* accelerates senescence and increases grain protein content when introgressed into wheat lines not carrying the gene represents a significant scientific breakthrough. For *OsNAP* reduced expression resulted in delayed senescence, an extended grain-filling period, and increased grain yield compared to wildtype plants [28]. Furthermore, recent studies showed that overexpression of *TaNAC-S*, a novel wheat NAC transcription factor, resulted in delayed leaf senescence and higher nitrogen concentration [156]. These examples suggest that NAC transcription factors can be essential for improving nutrient remobilization during senescence and hence the productivity of cereal crop plants.

## 6. Conclusions

NAC transcription factors are extensively implicated in the senescence process as judged from global gene expression analysis. Although most senescence-associated NAC proteins cluster in three NAC subfamily, they are also spread all over the phylogenetic NAC tree, and NAC genes with essential functions in senescence, such as JUB1 and VNI2, also map to less characterized subfamily of the NAC family. This is suggestive of a large, as yet unexplored role, of NAC genes in senescence. Local NAC-centered GRNs are emerging, and the mode of action of NAC genes and proteins in the networks is becoming apparent. This reveals that NAC transcription factors often integrate abiotic stress responses and ABA-hormone regulation with the age-dependent senescence process. Hallmarks of the senescence process, such as chlorophyll degradation, can be explained by the molecular mechanisms of NAC transcription factors such as *At*NAP and ATAF1. Future studies should further unravel the complicated GRNs connecting age-dependent and stress-induced senescence. In addition to the approaches which have already been used, omics strategies such as metabolomics as used for studies of *Pop*NAC154 and proteomics revealing aspects of protein levels, interactions and protein modifications should be used in the future. Structural data on the DNA binding NAC domain and the intrinsically disordered transcriptional regulatory domains will also be useful tools for expanding knowledge of the GRNs. The story of the *Gpc-B1* gene in wheat shows that NAC genes can be important factors in breeding of crop species for improved quality traits. Evolutionarily, the loss of the *Gpc-B1* gene in domesticated wheat was probably part of extending the growth season for this crop under temperate climate, *i.e.*, by delaying senescence. Conversely, the targeted re-introduction of the active *Gpc-B1* gene into hexaploid wheat appears to improve nutrient remobilization during grain filling. The story of *OsNAP* in rice also demonstrates that NAC genes can be targeted to obtain specific changes in lifespan control and nutrient remobilization in crops. Furthermore, the complexity of the regulation of senescence, associated also with stress regulatory processes, which comes out of the studies on the NAC gene family in Arabidopsis, clearly points to great potentials in exploiting the large variability in this gene family for targeted breeding efforts in crop plants, with the aim to increase or stabilize yields, improve nutrient contents of harvested seeds, and improve nutrient use efficiency. This will be of great value for future breeding of crops to ensure robust and high-yielding cultivars under the anticipated future more harsh growing conditions imposed by climate change.
